# A minimal metadata set (MNMS) to repurpose nonclinical in vivo data for biomedical research

**DOI:** 10.1038/s41684-024-01335-0

**Published:** 2024-03-04

**Authors:** Anastasios Moresis, Leonardo Restivo, Sophie Bromilow, Gunnar Flik, Giorgio Rosati, Fabrizio Scorrano, Michael Tsoory, Eoin C. O’Connor, Stefano Gaburro, Alexandra Bannach-Brown

**Affiliations:** 1 Roche Pharma Research and Early Development, Data & Analytics, Roche Innovation Center Basel, F. Hoffmann-La Roche Ltd, Basel, Switzerland; 2 Neuro-Behavioral Analysis Unit, Faculty of Biology & Medicine, University of Lausanne, Lausanne, Switzerland; 3 Group Legal Department, F. Hoffmann-La Roche Ltd, Basel, Switzerland; 4 Discovery, Charles River Laboratories, Groningen, the Netherlands; 5 Tecniplast S.p.A., Buguggiate, Italy; 6 Emerging Technologies, Comparative Medicine, Novartis International AG, Basel, Switzerland; 7 Behavioral and Physiological Phenotyping Unit, Department of Veterinary Resources, Weizmann Institute of Science, Rehovot, Israel; 8 Roche Pharma Research and Early Development, Neuroscience & Rare Diseases, Roche Innovation Center Basel, F. Hoffmann-La Roche Ltd, Basel, Switzerland; 9 QUEST Center for Responsible Research, Berlin Institute of Health at Charité–Universitätsmedizin Berlin, Berlin, Germany

**Keywords:** Research data, Biological models

## Abstract

Although biomedical research is experiencing a data explosion, the accumulation of vast quantities of data alone does not guarantee a primary objective for science: building upon existing knowledge. Data collected that lack appropriate metadata cannot be fully interrogated or integrated into new research projects, leading to wasted resources and missed opportunities for data repurposing. This issue is particularly acute for research using animals, where concerns regarding data reproducibility and ensuring animal welfare are paramount. Here, to address this problem, we propose a minimal metadata set (MNMS) designed to enable the repurposing of in vivo data. MNMS aligns with an existing validated guideline for reporting in vivo data (ARRIVE 2.0) and contributes to making in vivo data FAIR-compliant. Scenarios where MNMS should be implemented in diverse research environments are presented, highlighting opportunities and challenges for data repurposing at different scales. We conclude with a ‘call for action’ to key stakeholders in biomedical research to adopt and apply MNMS to accelerate both the advancement of knowledge and the betterment of animal welfare.

## Abstract (web summary)

Biomedical research is experiencing a data explosion, but this does not guarantee building upon knowledge gained from previous experiments. Without appropriate metadata, data can be wasted, especially in animal research. A minimal metadata set is proposed to enable data repurposing, aligning with ARRIVE 2.0 guidelines and making in vivo data FAIR-compliant.

## Main

Biomedical research is experiencing a data explosion, fueled by recent technological advancements that have accelerated data production capabilities. Data-rich multiomics approaches and high-resolution functional measures, such as multimodal imaging or recordings of physiology and behavior, are routinely being employed across the entire lifespan of model organisms in both health and disease states.

On the one hand, this new era presents a great opportunity to accelerate scientific understanding. On the other hand, the mere collection of vast amounts of data is not sufficient to ensure scientific progress if these data cannot be interrogated and reintegrated into the research cycle. One consequence of limited data sharing and poor transparency might be the need for repeated replication of prior findings, frequently without success^1–3^. These common practices result in a substantial waste of resources and missed opportunities for data repurposing. This topic is especially pertinent to research involving animals. Failures to replicate findings and missed opportunities for data repurposing undoubtedly lead to animal use that provides little or no new scientific progress and is cause for ethical concern. Thus, there is an urgent need to encourage and facilitate repurposing of nonclinical in vivo data in biomedical research.

In Europe and North America, legislation for animal experimentation in biomedical research focuses heavily on implementation of the 3Rs (see definition in Box 1), which encompasses the concepts of replacement, reduction and refinement^4^. The objective of the 3Rs is to ensure that animal experimentation achieves the highest level of welfare while minimizing burden through well-designed and reviewed animal research protocols and procedures. Yet, despite this robust regulatory framework, it is becoming increasingly clear that regulatory guidance protecting animal welfare standards alone is not sufficient to guarantee that research involving animals is minimized^5,6^.

To draw more awareness to concepts of validity, robustness and reproducibility (see definition in Box 1), the 3Rs principles have since been expanded to include the responsible use of animal research^7–9^. Open research practices, data sharing and FAIR (Findable Accessible Interoperable and Reusable; see also definition in Box 1) principles are complementary solutions that have been proposed to increase transparency and reproducibility^10^. Domain-specific solutions to assist researchers in creating datasets from their animal experiments have also been established. Examples include the Open Data Commons for Spinal Cord Injury and Traumatic Brain Injury^11–13^, and guidelines on the ‘Minimum Information about Animal Toxicology Experiments’ (MIATE)^14–16^. Meanwhile, the domain-agnostic and nonmandatory guidelines PREPARE^17^ and ARRIVE^18,19^ (see definition in Box 1) were also proposed as checklists for scientists when planning and reporting in vivo experiments, respectively.

In certain sectors, regulation mandates data sharing from studies involving animals and progress is being made to ensure that in vivo data can be repurposed with a view to generating virtual control groups (VCGs; see definition in Box 1). For example, in the EU REACH (Registration, Evaluation, Authorisation and Restriction of Chemicals)^20^, European Union (EU) biocides^21^ and EU plant protection products^22^, there is a legal requirement to share test and study reports from studies in animals that are used for registration purposes (see, for example, Article 62 in Regulation (EC) No. 1107/2009 (ref. ^22^)). Regulatory submissions to the US Food and Drug Administration (FDA) must adhere to the Standard for Exchange of Nonclinical Data (SEND), which requires presentation of data from nonclinical safety and toxicology studies in a consistent and machine-readable format. Finally, the recently concluded Innovative Medicines Initiative (IMI) eTRANSAFE (Enhancing Translational safety assessment through Integrative Knowledge Management) has further promoted guidelines and policies for the sharing of drug-safety-related data. The vision for this initiative is to improve translational safety assessments in drug development, including the potential for use of VCGs in nonclinical toxicity studies (see ref. ^23^ and more recently ref. ^24^).

Despite various regulatory frameworks, initiatives and guidelines, data sharing and repurposing within the field of biomedical research remains an exception rather than the rule^25–27^. Reasons for this limited progress may include domain-specificity of approaches, technical barriers to understanding FAIR data standards, a reluctance to share data, a lack of awareness of potential benefits and an absence of incentives for data sharing and repurposing.

One critical element necessary for data sharing and repurposing is to provide metadata (see definition in Box 1) that describe the raw or primary data (see definition in Box 1). Metadata are essential to make raw or primary data that are stored in data repositories (see definition in Box 1) FAIR. With appropriate metadata, researchers can effectively interrogate the underlying raw or primary data and realize the potential for repurposing. However, to our knowledge, a minimal metadata set (MNMS) for in vivo biomedical research that could be used in a domain-agnostic way (that is, across neuroscience, cardiovascular science, immunology and so on) has not yet been established. An ideal MNMS would build on existing guidelines for in vivo data reporting that are established for biomedical research, while also expanding their impact and applicability by opening the door toward effective data sharing and repurposing.

In this Perspective, with this need in mind, a working group of scientists from academia and private industry was formed to propose a MNMS to describe data generated from an in vivo biomedical research experiment. Additionally, we highlight opportunities, challenges and future actions required to support the adoption of MNMS in biomedical research with a view to ultimately enable data repurposing, the advancement of scientific knowledge and the betterment of animal welfare.

Box 1 Definitions of key terms **API**: an acronym that stands for application programming interface. An API is a set of protocols for communication and automated data transfer between two computer applications.

**ARRIVE**: the ARRIVE guidelines (Animal Research: Reporting of In Vivo Experiments) were originally developed in 2010 to improve the reporting of animal research. They consist of a checklist of information to include in publications describing in vivo experiments to enable others to scrutinize the work adequately, evaluate its methodological rigor and reproduce the methods and results^18^.

**Data repository**: a data repository is a structure consisting of one or more databases containing data for the purpose of analysis. Data repositories are used in business to provide a centralized source of information. A data repository may also be referred to as a data library or a data archive.

**Digital object**: a digital object is any kind of data that exists in a digital modality. A digital representation of a physical object or a process is also a considered a digital object.

**FAIR**: an acronym that stands for Findable Accessible Interoperable Reusable.

**Meta-analysis**: a meta-analysis is a statistical technique that combines findings from multiple independent scientific studies. In the clinical/preclinical context, meta-analysis is most often used to assess the effectiveness of interventions by combining data from several randomized trials.

**Metadata**: metadata are data on data (that is, information about the data), and contain descriptive and administrative information about the dataset. Examples include the project owner, title and persistent identifiers, as well as structural information about how the dataset was created. Further, machine readability of metadata is a high priority.

**OBI**: an acronym that stands for ontology of biomedical investigations. Community standard for scientific data integration. The OBI helps communicate clearly about scientific investigations by defining more than 2,500 terms for assays, devices, objectives and more.

**Ontology**: an ontology is a system of carefully defined terminology, connected by logical relationships and designed for both humans and computers to use.

**3Rs**: an acronym that stands for replacement, reduction and refinement. These are the guiding principles of animal research^4^.

**Raw data**: also known as primary or source data, raw data are data (for example, numbers, instrument readings, figures and so on) collected from a source that was not subjected to (1) processing, (2) ‘cleaning’ by researchers to remove, for example outliers and obvious instrument-reading errors, (3) any analysis (for example, determining central tendency aspects such as the average or median result) or (4) any other manipulation by a software program or a human researcher, analyst or technician.

Note that raw data provide a great deal of flexibility in terms of data repurposing, given that different questions can be asked from the original dataset that may not be possible after processing. However, raw data can be cumbersome to manage, and some preprocessing is often necessary to enable its useful interpretation. As such, ‘primary data’ refers to minimally processed data that provide the most flexibility and utility for additional analysis.

**Reproducibility**: here we refer to reproducibility broadly, to include both the stricter definition of reproducibility as ‘reproducibility of analysis’, referring to the re-analysis of an existing dataset, as well as ‘reproducibility of experimental findings’, which refers to the collection of new data in experiments as identical as possible to the initial experiment^40,41^.

**VCGs**: constitute digitally archived datasets encompassing both data and metadata, designed to optimize statistical models for predicting outcomes on the basis of quantifiable variables. Virtual controls act as a digital reference, simulating either the existing standard of care (that is, active reference control) or an absence of any intervention (for example, vehicle control). When an experimental animal cohort undergoes a specific intervention, these amassed digital data, in conjunction with predefined algorithms, forecast the potential outcome for that cohort in the absence of the said intervention. These predicted outcomes are termed ‘virtual controls’. Subsequently, the empirically observed outcomes postintervention are juxtaposed with these virtual control outcomes to ascertain effect magnitudes (adapted from ref. ^44^).

## Conceptual understanding of minimal metadata selection

Before outlining a MNMS for in vivo data, it is first essential to understand in more detail how metadata can aid the formation of a data repository and the decision to repurpose data, thus contributing to a reduction and replacement of animal use. Specific opportunities for data repurposing are discussed later in the manuscript (see ‘Benefits of adopting MNMS’ section).

**Fig. 1:**
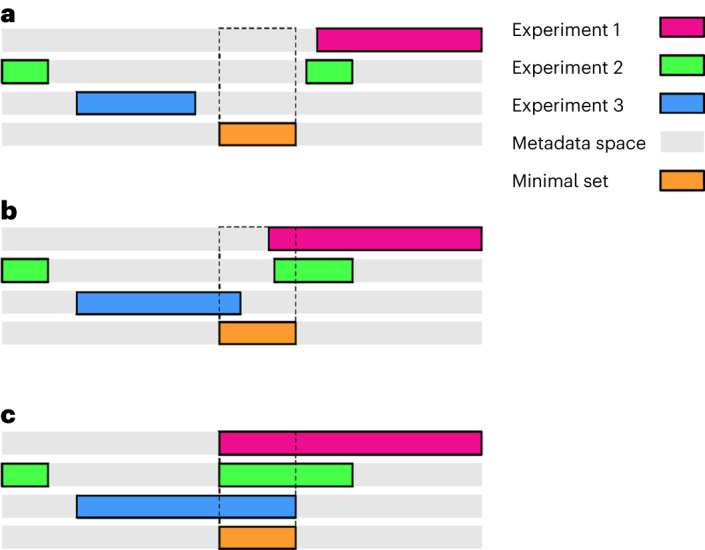
Three scenarios illustrating the relations between metadata sets from three different experiments. The extent of the metadata space is depicted as gray rectangles. The MNMS and its overlap with the three experiments is depicted as an orange rectangle, and an extending box with a dotted contour, respectively. **a**, Metadata do not overlap, leading to a worst-case scenario for data repurposing. The researchers cannot make any decision on the ability to repurpose data. **b**, Only a partial overlap is present between all three experiments. This scenario may incite researchers to fill in missing information with implicit knowledge, leading to detrimental consequences for the quality of data repurposing. **c**, All three experiments are associated with metadata that include the region occupied by the minimal set. In this case, the researcher can confidently operate a choice for repurposing the data.

In principle, the extent of metadata required to describe data generated from all biomedical research experiments covers an almost unlimited space. Practically, only a small portion of this space is needed (that is, necessary and sufficient) to effectively describe data from one experiment, and researchers can freely choose metadata sets that are fit for purpose. For illustration, we show a worst-case scenario (Fig. 1a) where metadata sets used to describe distinct experiments are disjointed and largely nonoverlapping. In this hypothetical scenario, the associated experimental data would reside in distinct regions of the metadata space. This segregation would hinder communication and interaction between different data sets. Therefore, the lack of metadata overlap would not allow the researcher to evaluate the potential to repurpose the data for their own needs.

In a more plausible scenario (Fig. 1b), a partial overlap between metadata sets can be found. However, critical metadata items required to describe the underlying data, and that are common to all the experiments, may still be missing. This scenario is typically encountered in the context of meta-analysis (see definition in Box 1) and systematic publication reviews. The absence of key metadata items can limit inference from such studies and is also detrimental for data repurposing. Specifically, a partial overlap of metadata may prompt researchers to resort to tacit knowledge for filling in missing information. This practice would lead to the aggregation of data collected either on incompatible sources (for example, the attempt to aggregate data from distinct mouse strains), or with discordant methodologies (for example, mice reared under different housing conditions), which would be inappropriate for data repurposing.

In the final scenario (Fig. 1c), aligning the metadata would yield a greater usability of the associated raw data. In this case, metadata from all three experiments would include a complete overlapping MNMS. Under our initial assumption, the experiments were originally logged with the metadata strictly necessary to reproduce the family of experiments they belong to. Adding the extra MNMS would not automatically extend the repurposing of the raw data that lay in distant regions of the metadata space. However, the presence of a complete MNMS would support the decision of the researcher as to whether include the associated raw data in a repurposing opportunity. Undoubtedly, the additional burden of logging the MNMS is eclipsed by the advantages that such a strategy offers in terms of data repurposing, including the potential for replacement and reduction of animal use, and reusage of critical data assets.

**Fig. 2:**
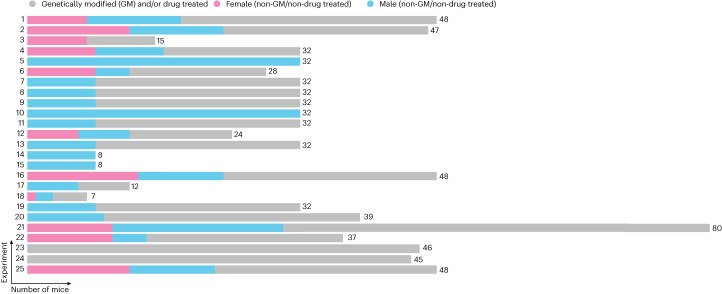
A practical example of using metadata to support the formation of a data repository. The schematic represents a real-world example of a repository of data from recordings of locomotor activity in mice, where all data are annotated with metadata. A total of 25 experiments are illustrated as separate rows, containing recordings from 828 individual mice. Filtering the database using metadata terms can quickly identify control, non-genetically modified and/or non-drug-treated male (blue, *n* = 219) and female (pink, *n* = 94) mice in each experiment. Data can be further segregated (for example, by strain, age and so on), enabling the experimenter to understand the potential for repurposing these data for their specific questions and needs.

As a more practical example for how metadata and database formation can support data repurposing, a schematic is shown of a data repository that was implemented at the Roche Innovation Center Basel (Switzerland) in 2022. This repository stores data from recordings of locomotor activity in mice obtained using a standardized protocol (Fig. 2). Given that data from each mouse are annotated with metadata, it is possible to quickly identify ‘control’ animals that were not subject to any pharmacological challenge and/or which were not genetically modified. Data from these animals can be quickly plotted and further interrogated by any Roche scientist with access to the database, thus supporting reuse of existing digital assets. With this simple example, one begins to appreciate how new questions can be asked of data repositories, while these questions were outside the scope of the original experiments stored within those repositories (for example, how does locomotor activity differ between different mouse strains and between sexes?). This approach takes on even more importance as the data repository continues to grow, supporting more complex meta-analyses. Having data stored in this way can facilitate the understanding of between-experiment variability and supports optimization of future experimental design. Moreover, establishing such a data repository annotated with metadata opens the door toward implementation of VCGs (this point is discussed further in ‘Benefits of adopting MNMS’ section).

## Key principles for the deployment of a MNMS

With the conceptual understanding of a MNMS in place, we next highlight key principles required for deploying a MNMS for in vivo research.

### FAIR

The applicability of the FAIR principles^28^ for diverse datasets has increased in the past decade. With the emergence of automated machine learning and artificial intelligence pipelines, emphasis is increasingly given to deploying FAIR principles to render data machine-actionable (that is, computational systems can find, access, interoperate and reuse data with no or minimal human intervention). Accordingly, metadata must foremost provide sufficient contextual information on the data. With regard to in vivo studies, granularity is optimally provided at the individual animal level and each digital object (see definition in Box 1) should be ‘findable’. This means that each animal should be assigned a unique identifier (that is, a uniform resource identifier (URI)) within the prospective data repository.

On a second level, metadata itself must also adhere to FAIR principles. To enable the interoperable aspect, a structure must be imposed on the metadata using a well-defined conceptual model to describe relationships and constraints between the different entities (for example, an animal or a study). This conceptual model enables a common understanding of metadata items and can be described using established standards to represent information within computer systems (for example, Resource Description Framework, Web-Ontology Language and JSON-Linked Data). To avoid variability and ambiguity, the metadata should consist of standardized elements such as controlled terminologies to ensure that these can be reused. For this purpose, adhering to existing ontologies per domain, if available, is strongly recommended (for example, SEND/CDISC or Ontology for Biomedical Investigations (OBI); see definitions in Box 1). In addition to having harmonized and standardized metadata, according to the FAIR principles, each metadata term should also be adequately defined, with a description of its use, and be given a unique identifier. Different synonyms of the term should also be captured. Collectively, this approach will lead to high quality and confidence in the metadata set provided.

### Integrity metric

Once a MNMS is agreed upon, checking the coverage and quality of the items reported in this minimal set would yield a ‘completeness score’ attached to each dataset, which would serve as an integrity metric. A completeness score associated to the metadata set is critical to enable a threshold-based decision concerning the rejection or inclusion of the associated raw data within a data repository. In addition, knowing the completeness of the metadata set would support researchers in assessing the generalizability of inferences that can be drawn from any repurposed data.

### Prespecification

In line with other best practices for reproducible research, the design of high-quality metadata structures should be prespecified. Prespecification avoids ‘post-mortem solutions’ that lead to both low-quality data reporting and an unwarranted confidence in the rigor of the methods adopted. Indeed, retrograde application of metadata structure is not guaranteed to succeed. For example, research environments are highly dynamic settings with often high rates of personnel turnover. In these situations, the task of recovering a comprehensive set of metadata based on a limited amount of metadata available becomes unsurmountable and error prone. For this reason, we strived to design a MNMS that emphasizes the prespecification of a limited number of actionable items. Limiting the number of prespecified metadata fields is critical to avoid placing an extra administrative burden on researchers.

### Provenance

Provenance and ownership of data are important aspects for a MNMS and are essential for curation in the context of large-scale usage of data and metadata sets. To enable identification of provenance and ownership, each data entry in a prospective repository must have the following operational metadata associated to it: creator of the record (which is also treated as a FAIR object with an assigned unique identifier), date of creation and date of modification. Finally, each of these terms should be given a definition to avoid potential ambiguity.

## The MNMS

The MNMS proposed here (Tables 1 and 2) builds on the ARRIVE 2.0 guidelines^19^. Specifically, the ARRIVE ‘Essential 10’ (Table 1) and the ‘Recommended Set’ (Table 2) served as the foundation for building the MNMS for biomedical research. These existing guidelines were selected because they are well known within the biomedical research community and are endorsed by peer-reviewed journals, which may facilitate future adoption of MNMS. Previous research on the ARRIVE 1.0 guidelines operationalized them into a list of over 100 items^29^, which can be difficult for authors to fully comply with; this extensive list also complicates the editorial staff’s task to check for compliance. With the revision of the guidelines in 2020, the ARRIVE 2.0 ‘Essential 10’ was put forward as the minimum information required for reporting of animal experiments^19^. Thus, we prioritized alignment of MNMS with the ‘Essential 10’, which would also help to minimize the workload for MNMS and thus further support its uptake. The MNMS proposed here would substantially contribute to making in vivo data from biomedical research FAIR compliant, in line with the original goals of the ARRIVE guidelines. Indeed, ensuring data from animal experiments is actionable through the adoption of MNMS further increases the impact of the ARRIVE guidelines. Below we further elaborate on the metadata terms included in MNMS. Definitions for specific terms used in the following section are provided in Box 2.

**Table 1:** The MNMS (ARRIVE 2.0 Essential 10)

ARRIVE topic-essential 10	MNMS	MNMS detailed	Data type	Existing ontology
Study design	Yes, but only start and end date of the in-life phase	Start and end date of the in-life phase	Date	ISO8601
Sample size	NA	NA	NA	NA
Inclusion/exclusion criteria	NA	NA	NA	NA
Randomization	NA	NA	NA	NA
Blinding	NA	NA	NA	NA
Outcome measures	Yes, extended, assay specific	Outcome measure (including any descriptive statistics if applicable, for example, average speed)	Controlled vocabulary	Multiple, assay specific per domain
		Unit of measurement per measure	Controlled vocabulary	UO, OBI
Statistical methods	NA	NA	NA	NA
Experimental animals	Yes, extended, all assays	Unique animal identifier	URI	NA
		Local identifiers/other IDs	String	NA
		Species	Controlled vocabulary	OBI, NCIt
		Strain (ILAR and short name)	Controlled vocabulary	ILAR^a^, MGI
		Sex	Controlled vocabulary	NCIt, CDISC/SEND
		Transgenic	Boolean	NA
		Genotype information	Controlled vocabulary	MGI
		Allele information	Controlled vocabulary	MGI
		Animal vendor (site and location) information	Controlled vocabulary	^a^
		Date of birth	Date	ISO8601
		Developmental stage	Controlled vocabulary	OBO Foundry
		Animal weight at start of experiment and unit	Number + controlled vocabulary	UO
		Severity grade of manipulation	Number	^a^
Experimental procedures	Yes, but focused on compound treatment; terms for procedures beyond require domain-specific alignment	Test substance (common name)	Controlled vocabulary	ChEBI, DrON
		Test substance (CAS number)	Number	NA
		Numerical dose	Number	NA
		Dose unit (ideally mg/kg or mM)	Controlled vocabulary	UO, OBI
		Vehicle composition	Controlled vocabulary	ChEBI
		Route of administration	Controlled vocabulary	OBI
		Administration method	Controlled vocabulary	OBI
Results	NA	NA	NA	NA

ChEBI, Chemical Entities of Biological Interest; CDISC, Clinical Data Interchange Standards Consortium; DrON, drug ontology; NA, not applicable; NCIt, National Cancer Institute thesaurus; OBO, Open Biological and Biomedical Ontologies; UO, units of measurement ontology.

^a^Further development needed to provide a formal ontology.

**Table 2:** The MNMS (ARRIVE Recommended Set)

ARRIVE recommended set	MNMS	MNMS detailed	Data type	Existing ontology
Abstract	NA	NA	NA	NA
Background	NA	NA	NA	NA
Objectives	NA	NA	NA	NA
Ethical statement	NA	NA	NA	NA
Housing and husbandry	Yes	Light cycle	Number	NA
		Testing location/research site	Controlled vocabulary	^a^
		Enrichment	Yes/no	NA

NA, not applicable (see main text).

^a^Further development needed to provide a formal ontology.

Box 2 Key terms related to structured metadata fields proposed in the MNMS **Administration method**: indicates the method that is used for exposure and excludes the route of administration.

**Administration route**: indicates a part of the body through which or into which a material entity has/is/will be introduced.

**Allele**: a variant form of the same gene at the same position, or genetic locus, on a chromosome.

**Animal supplier**: an organization site that supplies model animals (needs organization name and site).

**Genotype**: indicates the genotype of an organism. At its broadest level, genotype includes the entire genetic constitution of an individual. It is often applied more narrowly to the set of alleles present at one or more specific loci.

**Species**: a group of organisms that differ from all other groups of organisms and that are capable of breeding and producing fertile offspring.

**Strain**: a population or type of organism that is genetically different from others of the same species and shares a set of defined characteristics.

**Transgenic animal**: a model animal in which foreign DNA has been introduced using biotechnology. Foreign DNA (the transgene) is defined here as DNA from another species, or recombinant DNA from the same species that has been manipulated in the laboratory before being reintroduced.

### ARRIVE topics included with extended focus in MNMS

These topics include experimental animals, outcome measures and experimental methods. Details on experimental animals constitute the main aspect of MNMS, because insufficient or inaccurate reporting of these attributes is considered one of the main challenges for data reproducibility. Therefore, we propose that any prospective data repository assigns a unique identifier to each animal. This will enable not only FAIR datasets but, along with other potentially unique identifiers entered (for example, radio frequency identification (RFID) tags), also provide unambiguous identification of the animal record in the data repository and the associated datasets. The latter is especially important if an animal is included in more than one study, to avoid false duplicates and the introduction of artificial confounders of variability in any repurposed datasets.

With respect to the basic attributes for experimental animals, having the standardized Institute for Laboratory Animal Research (ILAR) name in addition to any short strain names or synonyms should be mandatory. As mentioned in ‘FAIR’ section, each controlled term for each terminology (such as strain) should have a unique identifier and a concise description that maps any existing synonyms. Strain information alone is insufficient because inbreeding over a long period of time may cause genetic drifts that might influence experimental outcomes. Therefore, precise source information is critical. For transgenic animals in particular, genotype and precise allele information is required, again using controlled terminologies and unique identifiers. For allele information, researchers must use IDs from Mouse Genome Informatics (MGI) if available. MGI is the authoritative source in the field of mouse genomics given that its nomenclature follows the rules and guidelines established by the International Committee on Standardized Genetic Nomenclature for Mice. Similarly, for strains and the strain identifier, an external reference ID (for example, the provider website entry or repository entry) can be used.

To indicate genotype and allele zygosity, using agreed conventions that restrict the available terms reduce the risk of ambiguities. The Jackson Laboratory provides such an example^30^. Furthermore, for multiple transgenic/mutant alleles additional care should be taken to unambiguously map the correct genotype to each allele in the sequence that would constitute the full genotype (for example, allele 1: Tg/+; allele 2: Tg/Tg).

The last essential immutable information is the date of birth of the animal. Due to current practices or difficulties in obtaining precise information, this information should be additionally marked as ‘precise’ or ‘approximate’ to indicate its reliability. A unified date format, explicit to the user during data entry, is recommended.

From the set of nonimmutable animal metadata, animal weight at the start of the experiment (with controlled vocabulary for the unit) is mandatory in the MNMS. Weight, together with animal age, may serve as a useful indicator of well-being when comparisons can be made with standard growth curves if they are known for a particular species or strain. Reporting on the standardized maximum severity grade of manipulation, per individual animal, is an important addition. This information may serve as a proxy for stress levels and suffering, and may explain deviations in the experimental data, allowing exclusion of the animal(s) from further analysis if necessary.

With regard to study-design terms, MNMS requests the experiment start and end dates (defined as the start and end of the in-life phase). These metadata can (1) signify which animals belonged to the same study; (2) facilitate the reuse of longitudinal data sets, where entries linked to body weight and age can change over the course of the experiment; and (3) ensure awareness of when the data were produced, which may be an important consideration in the case of factors such as genetic drift.

Since pharmacological manipulations are common in biomedical research, different forms of exposure to an active substance and for different purposes (for example, as a therapeutic, or to induce a specific condition like disease or transgene induction) are represented extensively in MNMS. To ensure accurate reporting of any test substance, the use of a unique test substance identifier, such as a Chemical Abstracts Service (CAS) number is necessary. Synonyms such as the common drug name, pointing to the same entity, can complement a digital record of the substance identifier and its existing ontologies. The numerical dose and the dose unit (for example, mg/kg or molar concentration) represent the substance dosing. Other important factors in the administration of a compound are the method of administration and the administration route (for example, intraperitoneally, per os and so on) and the vehicle composition. These metadata, including the dose units, must be defined through controlled terminologies or ideally through existing ontologies such as ChEBI^31^ according to the FAIR principles described previously.

### Recommended ARRIVE set topics included in MNMS

These include housing and husbandry, protocol registration and data access. From the ARRIVE 2.0 recommended set, we considered some attributes to be essential for reproducibility of experimental datasets and have therefore included them in MNMS. These attributes are the light cycle, denoted as number of light:dark hours in a 24-h day, dietary status, enrichment and testing location or research site. To avoid extending terminology to different diets or feeding schedules and enrichment types, which may be more reflective of specific experimental procedures and thus beyond the scope of MNMS, both dietary status and enrichment are represented with one of only two states (that is, fasted or fed/not fasted, and the presence of enrichment or not). Reporting the animal experimentation license permission in which animals were used is as an additional guardrail that ensures the reported dataset was generated using the stated procedures. The animal experimentation license permission additionally ensures that animal welfare standards are certified by the respective animal welfare authority. Last, but not least, data access in MNMS extends beyond the scope of the ARRIVE guidelines and constitutes a critical aspect for identifying dataset ownership. Each user is given ideally a unique identifier, such as the ORCID ID (Open Researcher and Contributor IDentifier). This identifier allows for logging any modifications to the data and metadata set via timestamps showing creation date and modification date. A data access and audit trail ensure data integrity and quality. To this end, any prospective user-facing system cannot be completely public but rather based on a registration and authentication service.

### ARRIVE topics not included in MNMS

These include study design, study sample size, experimental unit, inclusion and exclusion criteria, randomization and blinding. Previous attempts have shown considerable challenges when trying to implement topics pertinent to study design in a large-scale data repository^32^. These challenges multiply further when broadening the scope of the dataset for a more general-purpose repository that can enable data sharing between multiple stakeholders. For these reasons, several ARRIVE topics are explicitly not included within MNMS, because they do not support the core objective of data repurposing. More specifically, using the single-animal level for metadata (and data reporting) negates the need for reporting the sample size and experimental unit. Aspects of study design before experimental manipulations, such as the definition of groups being compared, randomization, blinding and inclusion and exclusion criteria are clearly important for reproducing the study itself. However, these topics add little value for repurposing because data will be reused in different studies with new randomization algorithms and selection criteria. Finally, yet importantly, nomenclature among different control groups is largely nonstandardized, posing another challenge^32^; therefore MNMS provides a set of objective criteria and guidelines for including an animal record from a control group.

## Benefits of adopting MNMS

**Fig. 3:**
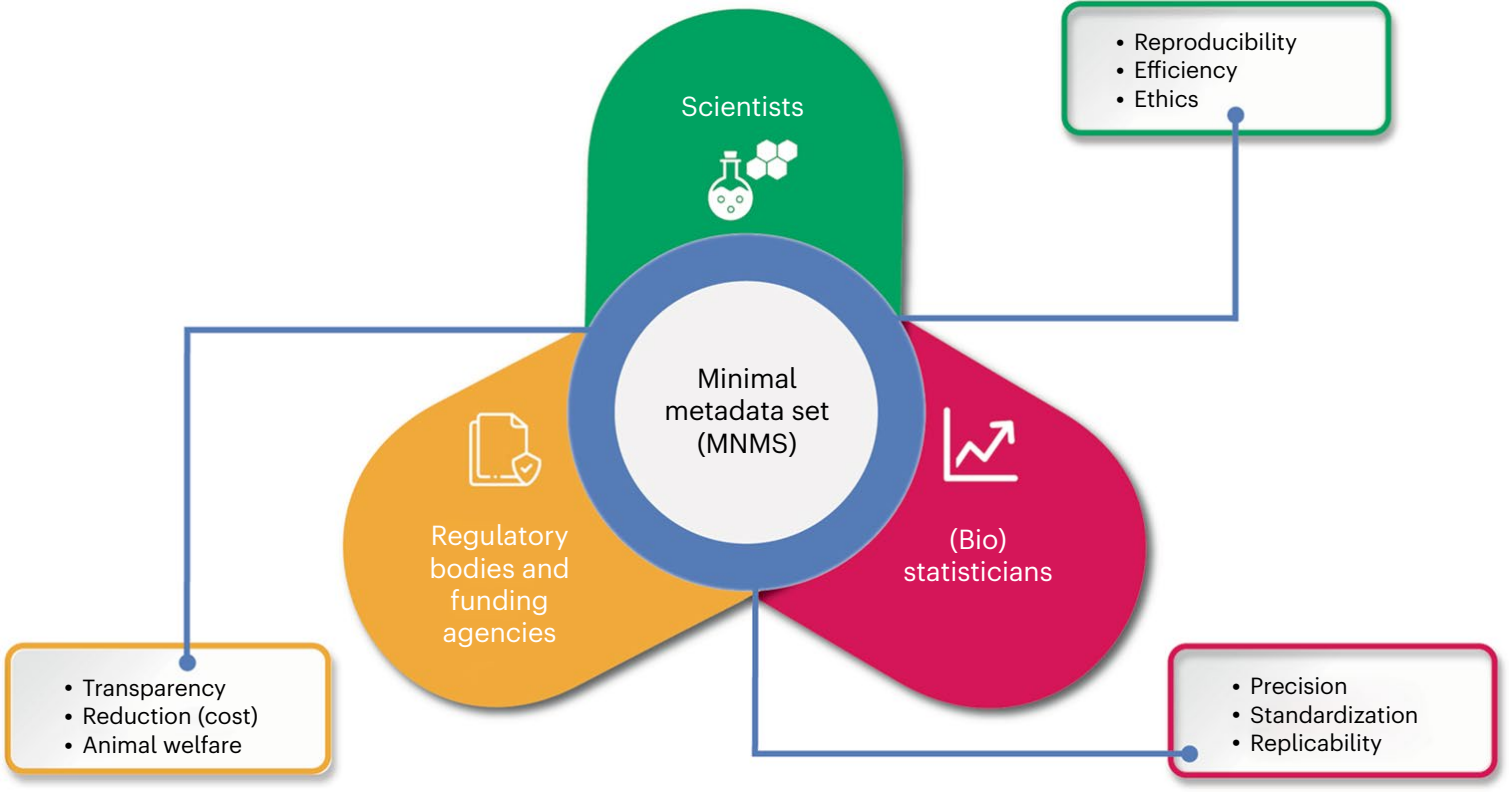
Advantages of implementing MNMS for key stakeholders. This figure illustrates the multifaceted benefits of MNMS across different stakeholders. For researchers, MNMS facilitates the creation of highly regulated and homogeneous experimental conditions, enhancing experiment repeatability, reducing resource use and addressing ethical considerations. Regulatory organizations and funding agencies benefit through the support of greater transparency, accountability and animal welfare, with MNMS enabling standardized experiments and reduced research costs. From the perspective of (bio)statisticians, MNMS increases the precision and replicability of statistical analyses by eliminating confounding variables, harmonizing data collection and ensuring a more regulated research environment. This comprehensive representation underscores MNMS’s role in advancing responsible, efficient and high-quality research practices.

With the MNMS outlined, in the following section we highlight the benefits that may come from adopting MNMS within the biomedical research community. These benefits include data repurposing, the generation of VCGs and facilitation of meta-research from in vivo studies. The benefits for various stakeholders in implementing MNMS are highlighted in Fig. 3.

### Repurposing

Reusing data obtained from animal experimentation serves a ‘repurpose’ function, and clearly falls within the 3R domains of replacement and reduction. The efficient description and retrieval of raw data enables their application for scopes beyond those intended during the original collection, therefore reducing the number of animals or, in some cases, replacing animals needed for novel experiments. An example of this approach was recently demonstrated by Fuochi et al., who reinterrogated data obtained by home-cage monitoring to gain new insights into mouse behavior^33^.

### VCGs

We envision that the use of VCGs, made possible from data sharing and data repositories that follow MNMS, would particularly benefit several stakeholders (Fig. 3). The opportunities and challenges for implementing VCGs were the focus of the IMI eTRANSAFE project, which has collected and analyzed drug safety and toxicology data from more than 60,000 rats, 1,300 dogs and 500 monkeys (see ref. ^23^ and more recently ref. ^24^). As further publications on VCGs emerge from this project, additional lessons and insights will undoubtedly further support the practical implementation of VCGs in the biomedical research field.

Our expectations are that VCGs would enable researchers to construct highly regulated and homogeneous experimental circumstances, which can improve experiment repeatability and reproducibility. Virtual animal controls may also improve research efficiency by saving time, costs and resources needed for animal experimentation. Furthermore, implementation of VCGs can address some of the ethical needs for responsible animal research by minimizing the number of animals required for biomedical studies.

For regulatory organizations and funding agencies, the deployment of VCGs can support objectives for greater transparency, accountability and animal welfare. Using VCGs to standardize animal experiments can assist regulatory agencies in monitoring and enforcing regulations, improving data transparency and accessibility, and reducing the number of animals utilized in research. Furthermore, adopting virtual animal controls can reduce research costs while also encouraging more ethical and responsible animal research. Successful implementation of this strategy would undoubtedly enhance public faith in regulation surrounding animal experimentation.

From the perspective of (bio)statisticians, VCGs can increase the precision and replicability of statistical analyses. Notably, VCGs can eliminate confounding variables, increase data quality and precision, harmonize data collection and reporting and create a more regulated and uniform environment for animal research. Collectively, these advantages can contribute to improve study results, data value and reliability of findings.

While VCGs may provide benefits for various stakeholders, we recognize that VCG implementation will not be without challenges in biomedical research. Careful consideration must be given to the construction of the VCGs. Should the control group be entirely virtual, or a mix of virtual and real data? If the latter, what is the optimal balance between virtual and real data? Statistical factors, such as selection criteria for virtual controls, data distribution considerations, variability and number of data points need to be considered. Ideally, the design and use of VCGs should be empirically validated, ensuring that experimental conclusions are consistent if VCGs are deployed in place of real data. Biological factors must also be considered, such as seasonal variability, the influence of individual experimenters or the potential for genetic drift, which may not be easily represented by VCGs. Thus, not all experiments will be able to benefit from VCGs and there will be continued expectation to run ‘real’ control animals when the use of VCGs is not feasible or sufficiently validated. Taken together, the implementation of VCGs must be carefully considered and validated, but their successful adoption may still provide major advantages to the biomedical research community.

### Meta-research in animal research

Meta-analysis of data from existing in vivo studies (within and beyond the scope of systematic reviews) provides a powerful tool to explore the impact of variations in experimental design and can reduce the need for further animal use. Pooling data from multiple studies on the same topic can increase the precision of pooled effect estimates. Meta-analyses of animal data are also used to inform the optimal design of experiments in a number of ways, including comparing performance and evaluating the necessity of outcome tests^34^, informing sample-size calculations^35^, refining the duration of experiments and humane endpoints^36^ and optimizing the choice of model induction technique^37^.

Although meta-analyses are an important approach to explore between-study heterogeneity^38^, they are resource intensive because of how data are currently reported in published research articles, typically as graphs or as group summaries (that is, group mean and variation). The graphical format poses challenges, as specialized tools are needed to first extract the estimated numerical summary information before further analyses can be conducted. The conclusions and accuracy of meta-analyses are therefore limited by the quality of primary data available. Adoption of MNMS as a reporting standard, for both control and experimental data, would provide a large step forward to improve the reporting of in vivo studies and facilitate the overall conduct of meta-analysis. The use of MNMS together with standardized repositories for data collection would also allow for complex meta-analyses that are currently prohibitive due to the absence of raw or primary data. Such work could generate novel scientific insights without additional use of laboratory animals.

A further benefit for adopting MNMS is that they can streamline the process for generating a richer set of metadata and insights with minimal additional effort, which can further support meta-analysis. One example to illustrate this logic is how to retrieve age-at-test, a metadata term that may be critical for meta-analysis, but which is not included in MNMS. The age-at-test can be obtained by intersecting the date-of-birth and the date of study start (both items included in MNMS) or the date of testing that may be embedded in the raw data file (for example, raw data timestamp). Therefore, MNMS serves as an additional layer of metadata that builds upon both the underlying dataset and any passive metadata generated through the data collection process. Intersecting and/or merging MNMS with the bare minimum of information derived from raw data enhances their value and utility. This approach also contains MNMS size to a minimum while maximizing its impact on data repurposing and the conduct of meta-analysis.

## Challenges for adopting MNMS

The concept of deploying a MNMS to enable the creation of structured data repositories and the opportunity to repurpose data is highly appealing from both a scientific (for example, incremental knowledge and new insights) and an ethical point of view (that is, replacing and/or reducing animal use). However, intrinsic to the concept of repurposing is the idea that a large user base is necessary to fully realize the opportunities for data reuse. Here we acknowledge that applying the repurposing concept on a large scale is not void of challenges.

### Adoption within the scientific community

Several initiatives have emerged to increase replicability and reproducibility, and to support reuse of data derived from in vivo studies^18,19,39^. Unfortunately, these initiatives and associated guidelines have not been routinely adopted across the biomedical research community. One explanation is that the efforts required to follow such guidelines, and the rewards gained from doing so, may not be of immediate interest to the data producer in the laboratory. Particularly within the field of discovery biomedical research, there may be limited perceived benefit to include accurately reported metadata and store data generated from highly customized ‘one-of-a-kind’ studies within a central repository. Historical practices within laboratories, combined with finite resources, may reflect the need for further change within the scientific community to recognize the importance of data sharing and the responsibilities of scientists to report their data.

To minimize the effort needed for providing MNMS and maximize their impact, we have proposed a MNMS that aligns with ARRIVE 2.0, as set of guidelines that are gaining increasing acceptance within the biomedical research community and are accepted by peer-reviewed journals. By providing a mandatory and minimal set of controlled terminologies to describe the data, the MNMS would overcome challenges of ‘free text’ entries required by ARRIVE, which can result in entries of variable quality that are not easily comparable between publications, or no entries at all.

Nevertheless, we acknowledge that ensuring ease of use alone is probably not sufficient to ensure uptake and implementation of MNMS across the biomedical research community. To further support uptake, additional tools, training and ultimately regulation will be required. Such tools may include the use of artificial intelligence to support identification and reporting of metadata from publications in standardized formats, and to highlight where certain metadata are not found and must be provided before publication. In addition, an ‘incentivizing’ system may be highly effective to support adoption of MNMS and data reuse. For example, data and metadata sets could be associated with quality metrics and a referencing system could be implemented. Thus, data and metadata sets that have supported reuse will receive a higher reference rate and provide recognition to the contributing scientists, research groups and their institutions.

A key step to increase data sharing and repurposing in biomedical research will be to implement controlled terminologies since such standards are not common in this field. The MNMS would provide a starting framework in which to implement these terminologies. The biomedical research space is large and diverse, which makes implementation of any standard challenging. However, this size and diversity of the research space also presents a tremendous opportunity to better leverage the knowledge being generated and further support the 3Rs. In that regard, it is valuable to highlight that progress in this direction has been made in other fields. For example, in the chemical industry, a legal requirement exists to share data from in vivo studies in reports that are used for registration purposes (see, for example, Article 62 (ref. ^22^)). Likewise, the FDA has implemented SEND for standardized reporting of nonclinical safety studies, while the recently concluded IMI eTRANSAFE project has advanced data sharing to improve translational safety assessments in drug development^24^.

### Constraining the experimental space

We believe that it is possible to identify a minimal set of metadata for animal experiments such that raw data can be repurposed across distinct families of experiments. However, this optimistic view is restrained by the extent of the experimental space and its relative metadata space (that is, all possible experiments across distinct disciplines).

This vast space does not allow the identification of a minimal set of metadata that guarantees a decision is always taken on the reuse of data from across diverse disciplines (for example, cardiology, neuroscience, oncology and so on). In such a massive task, MNMS would need to be expanded to an unmanageable size, therefore losing the appealing quality of being ‘minimal’. For example, while properties related to the animals and their housing may range over a limited number of dimensions (for example, genotype, age, weight, light cycle and so on), metadata from the domain of experimental manipulations cover a far more extensive space. This issue quickly emerged in the context of the present workgroup while searching for a consensus on the items to include in MNMS. Consensus was reached on several items belonging to the ‘Animal’ domain (for example, age, genotype and weight), while agreement quickly broke down when attempting to identify metadata pertaining to experimental manipulations. This fragmentation reflects the authors’ different research backgrounds, and, indeed, distinct domains are likely to occupy different regions of the metadata space.

To address the challenge given by the vast experimental space in biomedical research, we suggest that an a priori decision must be made concerning the range of experimental questions (and their associated outcome measures) that would be of interest to address in a repurposing initiative, such as in the generation of VCGs. Prespecifying and making explicit research questions (and outcome measures) will help impart a structure (Fig. 1c) to the metadata. This, in turn, will allow researchers to gauge the repurposing potential of the collected raw data in different disciplines and across institutions. Indeed, it is critical that multiple parties (for example, domain experts, data architects, funding agencies and so on) join in a concerted action to define the boundaries and bridges of these minimal sets of metadata and to make repurposing of data across disciplines a successful reality.

### Legal considerations for data sharing

The objective of deploying MNMS to facilitate data sharing and repurposing raises some important questions from a legal perspective. We believe that future initiatives to support data sharing would need to take at least the following ten topics into account.

#### Ensuring privacy of individuals

As discussed, an important aspect of metadata is to ensure provenance and ownership. The idea of incentivizing and recognizing contributors and users of data for repurposing would also require that individuals are associated with a FAIR identifier such as an ORCID ID. However, the ORCID ID is a code that can be linked back to an identifiable person, which is likely to make the code ‘personal data’ in some parts of the world. Contributors may therefore need to be given notice of how their personal data will be used and shared with others. Other measures may also be needed to ensure compliance with privacy requirements.

#### Control over who can access the data and for what purposes

To protect the value of the data, the data should be accessible only by those with genuine intent to conduct further research.

#### Minimizing disclosure of commercially sensitive information

Where requests for access are made by a competitor of the contributor, protective measures are likely to be necessary to protect such commercially sensitive information and to ensure compliance with competition law^40^. Potential options might include use of a third party to oversee and approve requests for access without involvement of the contributor.

#### Ensuring protection of proprietary data

For increasing the crowdsourcing user base, we propose keeping a focused scope on animals used only in control groups, where no proprietary product has been applied.

#### Fair access

Although companies are not forced to share data, it would be prudent to ensure that all the terms of access are fair, reasonable and nondiscriminatory (that is, those in a similar position should be able to access the data on equivalent terms).

#### Timing of access

Should data be available for others to access immediately, or is it reasonable to impose a time delay to protect the commercial interests of the contributor? Some existing tools already consider timing of access to information as a relevant feature (for example, see animalstudyregistry.org, which allows embargo time periods).

#### Ownership and rights of use

Contributors are likely to want to keep ownership of their data and only give recipients a right to use the data for certain uses (with limited rights to share with others). Where only control arm data are shared, the requestor will probably expect to own the results they generate using that data.

#### Liability

Requestors might reasonably expect that the contributor confirms that they have the right to contribute the data. However, contributors are likely to want the requestor to accept full responsibility for confirming that the data are suitable for the purposes for which they want to use it and to accept all liability associated with reuse of the data. Requestors might therefore need to thoroughly check the data to confirm that it is indeed fit for purpose, and this would need to be appropriately managed.

#### Price payable

Should the requestor be required to make a financial contribution toward the costs of generating data? If the decision is yes, then the guidance published by the European Chemicals Agency on data sharing^41^ may be a helpful reference point for how to calculate reasonable sums.

#### Ensuring the right to share

Contributors will need to ensure that their agreements with third-party contract research organizations (CROs) or research collaborators permit the sharing of the relevant data into any data-sharing scheme and the further reuse by third parties. This issue is particularly important where the sharing of the data is not a clear legal requirement and where there is an intent to charge third parties for access to the data (which may be viewed as commercial use of the results).

## Scenarios for MNMS implementation

Despite the recognized challenges discussed above, the following section highlights opportunities to leverage MNMS to support in vivo data repository creation and data repurposing across different in vivo research contexts, including behavioral core facilities in academia, CROs, pharmaceutical companies and in vivo research equipment providers.

### Behavioral core facilities in academia

Behavioral core facilities are increasingly common within academic research institutions, and they are well positioned to improve the quality of preclinical biomedical research by ensuring quality control measures^42^. Core facilities provide local users (typically principal investigators and laboratories from the university in which they are hosted) with access to a range of assays and equipment that facilitate functional in vivo research (see ref. ^43^ for more information). These assays and equipment are typically accepted as gold-standard approaches within the field. Experimental protocols applied are quite invariant and routinely repeated by different users who maintain the laboratory animals in the same facility and rearing conditions.

#### Opportunity

The unique position and resources of a core facility within academic research may allow data sharing and repurposing in a very straightforward and applicable manner. To support implementation of MNMS, several metadata fields (for example, strain, sex, age and so on) may be automatically drawn from the animal facility records with support from husbandry staff to provide information regarding rearing conditions (for example, diurnal light cycle). Core unit staff (if in place) can provide information regarding the experimental protocol (for example, apparatus setups if included in an extended metadata set) and the researcher can provide additional fields (for example, treatment, dose and route). The implementation of MNMS and supporting systems may be further expedited with support from local informatics services and infrastructure. In summary, behavioral core facilities in academia may be an excellent starting point for advocating and implementing MNMS.

### CROs

CROs provide access to in vivo experiments on a fee-for-service basis to a sponsoring client. Clients are typically private institutions, such as pharmaceutical or biotechnology companies, which may not have internal in vivo expertise or may wish to extend their in-house capacity. CROs typically offer a catalog of standardized in vivo assays that are accepted within the respective biomedical research field and follow local and international standards of regulation concerning animal experimentation. Many CROs also offer safety pharmacology and toxicology studies in animals that may be used to support regulatory submissions. In both the discovery and safety setting, customized in vivo assays can be developed and adapted to a client’s need. Notably, CROs are used to store data in a structured way in dedicated databases. Particularly for CROs that have implemented SEND, as required by the FDA for safety studies, a framework for the standardized, electronic representation of individual animal study data is already in place.

#### Opportunity

With the experience gained from data storage standardization within drug safety studies for SEND, a pull-through to in vivo studies for discovery biomedical research is an easy step for many CROs. There would be numerous benefits for adoption of MNMS and data repurposing in the CRO space for discovery research. First, by making control data or other experimental data (for example, from standard reference treatments) available for repurposing, sponsoring clients could avoid repetition of some experiments and choose to use VCGs instead of newly generated control data. This process would undoubtedly lead to a substantial reduction in the use of laboratory animals. Second, the exchange of data from client to CRO, and vice versa, should enhance the inherent robustness of a given assay, which would benefit both the CRO by emphasizing assay quality and the client by making robust decisions based on the assay result. For example, results may be reported that suggest a compound effect when it is rather driven by an aberrant control response. Having the ability to use larger VCGs would reduce this risk. Finally, the adoption of MNMS would support standardized datasets, which in turn can harmonize data to further align and optimize study designs. In the development of new medicines, time is of the essence. VCGs and optimized study designs will not only result in a reduction in the number of animals and ensure more ethical research, but it will also speed up the drug development trajectory and reduce costs, which is often a critical factor for smaller-sized biotechnology companies.

While numerous benefits are envisioned in the CRO space for adoption of MNMS and data repurposing, sharing data remains a point of attention. Restrictions on data use may be contained in the agreement between the CRO and the sponsoring client, and sharing data may require additional terms to be agreed (as discussed above in legal considerations for data sharing).

### Pharmaceutical research and development

While the vast majority of experimentation in pharmaceutical research and development (Pharma R&D) is undertaken in non-animal studies, animal research remains an important component to discover new biology and treatment opportunities, and to predict the efficacy and safety of new medicines before entering human trials. Within a single pharmaceutical company, multiple drug discovery programs may be running simultaneously, often over diverse geographical locations and via internal efforts and/or with external partners including academic collaborations, consortia or CROs. Research programs can last for years, or even decades, as is the protracted timeline typical for developing new medicines. In this context, FAIR data storage and the need to avoid continuous data replication of historical data is of paramount importance to ensure research progress and optimize insights discovery.

#### Opportunity

To address the challenges that are particular to Pharma R&D, a standardized approach to information technology architecture and data management is critical. Adopting MNMS would facilitate in vivo data repurposing, while open standards and application programming interfaces (APIs; see definition in Box 1) would facilitate interoperability between different systems, enabling researchers to work more efficiently and collaboratively. By creating a domain-agnostic platform integrated with animal management software, researchers can make their data more accessible to others, leading to greater collaboration and innovation. This data accessibility would allow researchers to build upon each other’s work and combine data from different studies to address new research questions.

To support implementation of MNMS into Pharma R&D, a multidisciplinary team consisting of data scientists, scientific researchers and laboratory animal specialists is essential for effectively implementing a global standardized approach. Collaboration between these fields can help ensure that the data management strategy is comprehensive and aligned with company research goals and requirements. Data scientists can contribute their expertise in data modeling, analytics and visualization to develop a common data model that can be used across different systems. Scientific researchers can provide input on the specific biomedical needs and objectives, as well as identify potential research questions that can be addressed through data repurposing. Laboratory animal specialists can provide insights into the operational challenges of working with different systems and help ensure that the strategy is compatible with local and global animal welfare regulations. Importantly, senior managers within Pharma R&D can recognize the importance of such initiatives and support their resourcing to ensure successful and timely implementation.

This collaboration model can lead to more efficient and accurate research outcomes, ultimately benefiting scientific progress, the development of new medicines and animal welfare. Standardization in data management can also improve data quality and consistency, which is essential for regulatory compliance and data sharing initiatives. By implementing a comprehensive approach that addresses the challenges of working with multiple in vivo systems, researchers in Pharma R&D can repurpose and combine data from different studies, leading to greater insights and discoveries.

### Animal research software and/or hardware developers and providers

Animal research is enabled by a rich ecosystem of hardware and software, including colony animal management software and laboratory information management systems, tools used in the laboratory for various measurements (for example, heart rate monitors or imaging devices) and software that enables biospecimen analysis and data visualization (for example, image analysis or statistical tools).

#### Opportunity

By incorporating MNMS and especially harmonized vocabulary terms into their platforms, businesses engaged in providing experimental equipment and software for in vivo research can greatly contribute to improving data sharing and repurposing. Particularly important in this context are companies that offer animal management software, which can provide users with access to MNMS and create and support software interfaces to push MNMS to other systems involved in data collection and analysis. Likewise, companies that support experimental data collection can ensure that MNMS are embedded into raw data to ensure seamless analysis and ease of integration when leveraging different commercially available systems. Notably, to incorporate MNMS in their products, commercial businesses must ensure that the same controlled vocabulary is used throughout data collection, management and analysis. A standardized approach would facilitate researchers’ access to, comparison of and reuse of data created across many platforms and studies.

By adopting MNMS, commercial system providers can contribute substantially to the scientific community by making important information readily accessible for a variety of purposes, including grant applications, academic publications, animal license applications and patent filings related to animal research. In addition to enhancing the quality and supporting reproducibility of research, this advance would further encourage ethical considerations and ultimately facilitate the process of animal-data management and repurposing.

## Concluding remarks and future directions

We have proposed a FAIR-compliant MNMS for in vivo studies in biomedical research. The MNMS that we propose, if adopted more widely, would provide multiple benefits. First, MNMS builds upon existing initiatives (for example, guidelines such as PREPARE and ARRIVE) to increase transparency in data generation. Transparent reporting of methodology and data required to replicate analyses is a fundamental step for enabling and ensuring reproducibility. MNMS is a tool that supports transparent reporting.

Second, implementing MNMS would facilitate data sharing, ideally through large publicly accessible data repositories. In turn, this would enable data repurposing, with just one of many examples highlighted being the generation of VCGs. The use of VCGs is a currently underexploited opportunity that could substantially reduce the number of animals used in biomedical research when applied at scale.

Taken together, we believe that the deployment of MNMS alongside existing initiatives (ARRIVE) represents the next frontier for advancing the ethical use of animals in research. Therefore, we advocate for its use and propose strategies for further development of MNMS and uptake among research stakeholders. While there will be challenges for MNMS implementation, lessons for successful in vivo data sharing can be learned from other fields including the chemical industry and drug safety testing.

To advance MNMS implementation, we propose an incremental and collaborative approach to refinement, testing, validation and implementation. A logical first step would be to pilot their use with the primary stakeholders (Fig. 3). This step could involve refining MNMS with co-creation and community-consensus communication processes between participants from each of the key groups (scientists, regulatory bodies and (bio)statisticians) across academic, pharmaceutical and contract research sectors, to identify further potential barriers and enablers to implementation. A technique such as a modified Delphi allows for community input and consensus when deciding on adding and refining other terms that are critical in specific fields, such as the importance of health status or animal microbiomes. Concurrently, additional cross-disciplinary efforts are required to create and refine comprehensive legal frameworks and policies for data sharing across stakeholders from multiple industries. These efforts could focus on topics such as the following: joint development of tools to support integration with existing data infrastructure; development of new documentation and education to support uptake; and integration with existing initiatives in 3Rs. We propose these activities are run in iterative rounds to align viewpoints within this diverse stakeholder group more rapidly.

The open conduct of multiple collaborative stakeholder activities will be critical for developing proof-of-concept exemplar projects, white papers or recommendations that can support integration of MNMS into existing cross-industry 3Rs initiatives. Examples of these initiatives include protocol registration (for example, animalstudyregistry.org and preclinicaltrials.eu) and initiatives to improve experimental design quality and reporting standards for animal experiments (for example, PREPARE and ARRIVE guidelines). In this context, policy makers, regulatory agencies and funding bodies will have a fundamental role in supporting adoption of MNMS within the wider research setting.

We highlight the need for further strategies that can support adoption and implementation of MNMS for data sharing. Obtaining insights on what tools the research community currently uses (for example, Research Data Alliance initiatives or domain ontologies and vocabularies), and how MNMS can best align and integrate with these tools, will enable the development of new strategies to tackle barriers to adoption of MNMS and facilitate data sharing. For example, additional documentation may be required for nontechnical users on how to use MNMS within their existing workflows. MNMS may also be further refined to reduce unnecessary overlap or redundancy in the workload required by primary stakeholders. A critical need is likely to be the harmonization of terminologies and controlled vocabularies, which could support MNMS adoption by key stakeholders, including companies providing scientific research equipment and software to in vivo researchers across sectors. We recognize these companies as a key enabler to facilitate uptake of MNMS and data sharing. Indeed, the private industry sector has already made considerable progress in the advancement and implementation of controlled vocabularies, and close collaboration between private and public institutions is likely to accelerate progress in this field.

With feedback from the primary stakeholders, a roadmap to MNMS dissemination can be put in place, probably moving from local to global use in a stepwise, incremental manner and ensuring alignment between these efforts at each stage. Dissemination activities could include hosting workshops to showcase MNMS functionality with exemplar projects; outlining the impact MNMS on research outcomes for each stakeholder group; developing targeted marketing materials, such as infographics to highlight the benefits of MNMS; producing educational materials and documentation to support training efforts on how to effectively use MNMS; and performing pilot experiments to demonstrate the utility of MNMS in common settings. These strategies can accelerate the uptake and use of this tool into existing workflows and increase awareness of the benefits of MNMS for a broad audience.

Biomedical research stands at a critical juncture, where there is great potential to generate new insights, owing to data-rich technologies that can now be rapidly deployed and at relatively little cost. However, in the absence of tools to effectively reintegrate the vast quantities of data generated into the research cycle, researchers face a situation of massive resource inefficiency with minimal scientific gain. This issue is particularly concerning in the context of research with animals where researchers are committed to follow the 3Rs that also recognize the need for responsible use of animals. A first and necessary step toward effective repurposing of data from in vivo studies in biomedical research is to ensure that raw data are effectively described with metadata. The MNMS that we propose here aligns with existing guidelines for reporting in vivo studies and, if adopted, would provide an important step toward advancing scientific knowledge and ensuring the continued ethical use of animals in biomedical research.
